# Nanometre-Scale Biosensors Revolutionizing Applications in Biomedical and Environmental Research

**DOI:** 10.3390/bios13110969

**Published:** 2023-11-06

**Authors:** Pik Kwan Lo

**Affiliations:** 1 Department of Chemistry and State Key Laboratory of Marine Pollution, City University of Hong Kong, Tat Chee Avenue, Kowloon Tong, Hong Kong SAR, China; peggylo@cityu.edu.hk; 2 Biotech and Health Care, Shenzhen Research Institute of City University of Hong Kong, Shenzhen 518057, China

Driven by the convergence of nanotechnology, biotechnology, and materials science, the field of biosensors has witnessed remarkable advancements in recent years. These miniature devices can detect specific targets by leveraging recognition receptors, and subsequently translate biomolecular interactions into measurable signals. The advent of nanometre-scale biosensors has opened new avenues for the application of these devices in various domains, including medicine, environment, food, and biomedical research. One of the key advantages of nanometre-scale biosensors lies in the availability of a wide array of recognition elements, such as antibodies, enzymes, nucleic acids, peptides, and carbohydrate-binding proteins. These elements can be easily synthesized and integrated into biosensor platforms, enabling the transformation of molecular binding events into various physical signals, including electronic, optical, magnetic, and mass changes. This versatility allows for qualitative and quantitative analysis of a broad range of biomolecules, proteins, DNAs/RNAs, biomarkers, metal ions, microorganisms, and toxin pollutants.

In this Special Issue, Moabelo et al. (contribution 1) reported the development of a rapid and specific biosensor for the detection of retinol-binding protein 4 (RBP4), a potential biomarker for the early diagnosis of type 2 diabetes mellitus (T2DM). The biosensor utilizes gold nanoparticles (AuNPs) as the sensing platform and employs a colorimetric detection approach. The retinol-binding protein aptamer (RBP-A) is immobilized on the surface of the AuNPs, enabling nanoparticle stabilization. RBP4 binds to the RBP-A, causing detachment from the AuNPs and resulting in the aggregation of the nanoparticles upon the addition of sodium chloride (NaCl). This aggregation leads to a visible color change in the AuNP solution. The developed assay provides a test result within 5 min and demonstrates a limit of detection of 90.76 ± 2.81 nM. The study highlights the advantages of using aptamers in biosensing applications, such as their high specificity, selectivity, low molecular weight, and ease of production. The colorimetric approach based on AuNPs offers simplicity, rapid response, and high sensitivity, making it suitable for use in point-of-care testing (PoCT) and resource-limited settings. This study provides a promising approach for the detection of RBP4, which can aid in the early diagnosis of T2DM.

In their research, Cui et al. (contribution 2) applied a microfluidic live-cell immunoassay, integrated with a microtopographic environment, in order to investigate intercellular interactions in different tumor microenvironments. The platform allows the coculturing of immune cells and cancer cells on tunable substrates and the simultaneous detection of different cytokines. It also enables the investigation of migration behaviors of mono- and co-cultured cells on flat and grating platforms, revealing topography-induced intercellular and cytokine responses. The study employs a microbead-based sandwich assay for on-chip cytokine monitoring and achieves precise quantification with a low sample volume and short assay time. The authors validate the biocompatibility of the co-culture strategy and compare the immunological states of different cell types on different substrates. The integrated microfluidic platform offers an efficient and precise approach for on-chip cytokine detection, eliminating manual sampling procedures and enabling continuous cytokine monitoring. The findings have implications for early immune diagnosis, personalized immunotherapy, and precision medicine. This study presents a novel microfluidic platform that enhances our understanding of intercellular interactions and cytokine secretion, providing valuable insights for the development of optimized diagnosis and treatment strategies in various fields. 

In this Special Issue, Lau et al. (contribution 3) demonstrated the design and development of small-molecule fluorescent probes, named DB-TPE, for the selective binding and detection of RNA sequences containing CAG repeats. These repeats are associated with neurodegenerative diseases such as Huntington’s disease. The researchers modified a bis(amidinium)-based binder, known for its selective targeting of CAG RNA, and incorporated an environment-sensitive fluorophore to create a turn-on fluorescent probe. The probe exhibited a significant 19-fold increase in fluorescence upon binding to a short CAG RNA sequence. The binding and fluorescence response were found to be specific to the RNA secondary structure with A·A mismatches. This study suggests that the DB-TPE fluorescent probe shows promise for application pathological studies, disease monitoring, and the diagnosis of neurodegenerative diseases linked to expanded CAG RNA repeats.

In this Special Issue, Monteiro et al. (contribution 4) utilized a sensitive and selective label-free photoelectrochemical (PEC) immunosensor for the detection of cardiac troponin I (cTnI), a biomarker of myocardial infarction. The immunosensor platform is based on a fluorine-doped tin oxide (FTO)-coated glass photoelectrode modified with bismuth vanadate (BiVO_4_) and sensitized by an electrodeposited bismuth sulfide (Bi_2_S_3_) film. The PEC response of the platform is enhanced in the presence of Bi_2_S_3_. The cTnI antibodies (anti-cTnI) are immobilized on the platform surface to create ananti-cTnI/Bi_2_S_3_/BiVO_4_/FTO immunosensor. When the immunosensor is incubated in cTnI solution, it inhibits the photocurrent generated by ascorbic acid, enabling the detection of cTnI. The immunosensor exhibits a linear relationship between the photocurrent and the logarithm of cTnI concentration within the range of 1 pg mL^−1^ to 1000 ng mL^−1^ The performance of the immunosensor is validated using artificial blood plasma samples, showcasing its successful application for cTnI detection with high recovery values. This study shows the potential of the developed PEC immunosensor for the sensitive and selective detection of cTnI, which can aid in the early diagnosis of myocardial infarction.

In this Special Issue, Zhang et al. (contribution 5) presented a new approach for the detection of silver ions (Ag^+^) using a minidumbbell DNA-based sensor (M-DNA). Silver ions are common heavy metal pollutants with potential health risks. The M-DNA sensor is designed with an 8-nucleotide minidumbbell structure containing a unique reverse wobble C·C mispair, which serves as the binding site for Ag^+^. The sensor demonstrates high accuracy in detecting Ag^+^ in real environmental samples, with a detection limit of 2.1 nM. The M-DNA sensor offers advantages such as fast kinetics and easy operation due to the use of an ultrashort oligonucleotide. This new DNA structural motif provides a promising platform for the development of on-site environmental Ag^+^ detection devices. The study contributes to the field of nanometre-scale biosensors and their applications in environmental monitoring, particularly in the field of heavy metal detection. 

This Special Issue contains several review articles highlighting different aspects of biosensors and their applications in various fields. These articles provide valuable insights into the development and utilization of nanometre-scale biosensors for diverse purposes. Chen et al. (contribution 6) emphasize the significant potential use of electrical impedance biosensors, integrated with handheld and bench-top microfluidic technologies, in various biological sensing applications. These biosensors enable easy operation by personnel without requiring specialized training. The use of microfluidics enhances sensitivity and analytical capabilities, reduces reagent consumption and analysis time, and improves reliability and efficiency through automation. Impedance biosensors offer extensive information about cell properties, eliminating the need for fluorescent labeling or other treatments, and serving as a cell fingerprint for identification and characterization. Lam et al. (contribution 7) summarize the strategies employed in Capture-SELEX, a modified SELEX method, for detecting and characterizing small-molecule-aptamer interactions. They discuss the development of aptamer-based biosensors for diverse applications. The advantages of aptamers over antibodies are highlighted, including thermal stability, low cost, chemical modification capabilities, and ease of generation. Aptamers exhibit selective and tight binding with a wide range of targets, including proteins, cells, microorganisms, and small-molecule contaminants. The article also addresses challenges associated with the Capture-SELEX platform and biosensor development, such as false-positive results and conformational changes of target molecules. Wen et al. (contribution 8) highlight the versatility of metal–organic frameworks (MOFs) in constructing single- or multi-emission signals for analytical purposes. They discuss the engineering and tailoring of photonic units within MOFs, originating from metal nodes, linkers, or guest molecules, to achieve diverse luminescence signals. MOFs show promise when applied to surface-enhanced Raman spectroscopy (SERS) methods, acting as substrates for signal enhancement or forming composite substrates by encapsulating metallic nanomaterials. The tunable properties, high surface area, controllable pore size, and sieving effect of MOFs contribute to improved detection selectivity, sensitivity, stability, and reproducibility. The potential of MOFs for multiplexed detection, integrating multiple signals into a single optical nanoprobe for ratiometric and multimodality measurements, is also discussed. Koyappayil et al. (contribution 9) highlight the recent trends, advances, prospects, advantages, and limitations of using metal nanoparticle-decorated 2D materials in electrochemical biomarker detection. Techniques such as electrochemical impedance spectroscopy (EIS), cyclic voltammetry (CV), differential pulse voltammetry (DPV), and square wave voltammetry (SWV), which possesses uses in biomarker detection systems, are discussed. The role of gold nanoparticles (AuNPs) on 2D platforms in enhancing signals and anchoring antibodies for biomarker detection is also described. Deng et al. (contribution 10) discuss strategies for the preparation and modification of semiconducting polymer dots (Pdots). Pdots have received significant attention due to their favorable photophysical properties and versatility, including large absorption cross-section, high brightness, tunable fluorescence emission, excellent photostability, biocompatibility, and ease of modification. They present recent advancements in the use of Pdots as optical probes for analytical detection and clinical applications in biosensing and disease diagnosis.

This Special Issue aims to showcase cutting-edge developments and innovative approaches that are helping to shape the field of nanometre-scale biosensors. By sharing knowledge and fostering collaboration, our goal is to accelerate progress and pave the way for practical applications that benefit society as a whole.

